# Adulthood Earnings Trajectory and 10-year Memory Decline: Findings from Administrative Earnings Data

**DOI:** 10.1093/geroni/igaf122.3525

**Published:** 2025-12-31

**Authors:** Calvin Colvin, Katrina Kezios, Samuel Swift, M Maria Glymour, Adina Zeki Al Hazzouri

**Affiliations:** Columbia University, New York, New York, United States; Boston University, Boston, Massachusetts, United States; University of New Mexico Health Sciences Center, Albuquerque, New Mexico, United States; Boston University, Boston, Massachusetts, United States; Columbia University Medical Center, New York, New York, United States

## Abstract

Financial resources are associated with cognitive functioning in older age, but prior studies do not characterize the dynamic trajectory of lifecourse financial status. Here, we examine the relationship between adulthood earnings trajectories and memory decline in older age using data from Health and Retirement Study participants linked to Social Security Administration earnings records. We included participants with any earnings data when 25 to 59 years old and ≥1 memory assessment on/after age 59 (N = 10,566). Memory was assessed using immediate and delayed 10-word recall or, for those unable to complete these tests, a proxy’s interview. We categorized age 25-59 earnings using group-based trajectory modeling, separately for each birth cohort and gender. Using confounder-adjusted linear mixed models, we examined the association of earnings trajectory with 10-year memory decline. The median age at first memory assessment was 60 (IQR: 59-62) years. Across birth cohorts and gender, participants with the highest earnings over adulthood had the slowest decline in memory. For example, among men in the earliest birth cohort (1931-1941), those with high initial earnings that increased over time had an annual rate of memory decline of -0.029 standard units (95% CI: -0.032, -0.026). In comparison, those who maintained low earnings experienced faster declines (βLowxTime = -0.012, 95% CI: -0.016, -0.007), corresponding to an annual rate of decline of -0.041 standard units (95% CI: -0.044, -0.037) Associations generally followed a dose-response pattern. These findings add to the growing literature showing relationships between financial resources and cognition. More work is needed to identify underlying mechanisms.

